# The Role of DAPK1 in the Cell Cycle Regulation of Cervical Cancer Cells and in Response to Topotecan

**DOI:** 10.7150/jca.66492

**Published:** 2022-01-01

**Authors:** Khayal Gasimli, Monika Raab, Sven Becker, Mourad Sanhaji, Klaus Strebhardt

**Affiliations:** 1 Department of Gynecology, School of Medicine, Goethe University, Theodor-Stern-Kai 7, 60590 Frankfurt, Germany.; 2 German Cancer Consortium (DKTK), German Cancer Research Center, Partner Site Frankfurt am Main, Frankfurt, Germany.

**Keywords:** cancer, cell cycle, protein kinases, death-associated protein kinase (DAPK1), polo-like kinase 1 (PLK1), topotecan

## Abstract

Cervical cancer is one of the most serious health conditions, with nearly 500,000 women developing the disease each year worldwide. At present, the treatment of recurrent cervical cancer remains largely ineffective, and efforts in cancer drug development are currently focused on critical serine/threonine kinases, such as death-associated protein kinase 1 (DAPK1) and polo-like kinase 1 (PLK1). In the current study, we aimed at exploring the cell cycle roles of DAPK1 and PLK1 in cervical cancer cells. To achive this goal, we used multiple methods including western blotting and assays for studying kinase activity, apoptosis, cell cycle, cell proliferation, immunofluorescence and proximity ligation. The present study demonstrated that, in cervical cancer cells, the enzymatic activity of DAPK1 was regulated in a cell cycle-specific manner. NIMA-related kinases, CDKs, PLKs and Aurora kinases regulate the function of centrosomes by orchestrating the separation of chromosomes during cell division. The present study added DAPK1 to this group of protein kinases due to its localization at centrosomes during mitosis. It was shown that DAPK1 was autophosphorylated at Ser308 in the G_2_ phase and during mitosis. From prophase to metaphase, the colocalization of PLK1 and DAPK1 at centrosomes was observed. Furthermore, the interaction of both these kinases could be demonstrated using proximity ligations assays and immunoprecipitations. DAPK1 was found to be a substrate of PLK1. Topotecan is an effective drug used for the treatment of cervical cancer. Therefore, the current study examined the role of DAPK1 in topotecan-induced cervical cancer cell death, and it was identified that RNA interference-based silencing of DAPK1 decreased the apoptotic effect of topotecan. Thus, these findings suggested that DAPK1 could be a biomarker and a potential target for the response to topotecan during the therapy of patients with cervical cancer.

## Introduction

Cervical cancer is the fourth most common female disease, and >500,000 woman are diagnosed worldwide with this disease every year 1. Moreover, this malignancy results every year in >300,000 deaths worldwide and is predominately caused by high-risk subtypes of the human papillomavirus 1. The therapeutic approach for the treatment of cervical cancer depends on the extent of the disease and is based on radical hysterectomy or chemoradiation, or a combination of both 1. At present, there is no standard chemotherapy regimen for patients with advanced or recurrent cervical cancer. Thus, clinicians and researchers continue to evaluate and compare different chemotherapy regimens for these patients.

Cisplatin is a commonly used chemotherapy drug for the treatment of cervical cancer 2. For adjuvant or neoadjuvant chemotherapy of locally advanced cervical cancer types and the treatment of metastatic or recurrent diseases, a range of novel agents (such as gemcitabine, the angiogenesis inhibitors bevacizumab and pembrolizumab, and nivolumab and cemiplimab that inhibit the immune checkpoint programmed cell death 1 protein) are currently being tested in clinical trials, with results from these studies being eagerly awaited 3. A recent phase III clinical trial reported that women with advanced cervical cancer treated with a combination of cisplatin (Platinol^®^) plus topotecan (Hycamtin^®^) chemotherapy had improved prognoses compared with women treated with cisplatin alone 4.

Targeting non-structural components of the cell cycle, including protein kinases and kinesins that serve prominent roles in multiple key signaling pathways, is a promising strategy for cancer therapy 5. The success associated with the clinical use of kinase inhibitors has thus inspired considerable efforts to develop selective small-molecule drugs targeting cell cycle-related kinases for cancer therapy 6, 7. Death-associated protein kinase 1 (DAPK1) is a member of the family of Ca^2+^/calmodulin (CaM)-regulated serine/threonine protein kinases. The DAPK gene is conserved during evolution from invertebrates, including *Caenorhabditis* (*C.*) *elegans*, to chordates and mammals 8. In humans the DAPK gene maps to chromosome 9q21.23. DNA methylation is a crucial epigenetic modification in mammalian cells. In particular, in different types of human cancer the inactivation of certain tumor-suppressor genes is due to hypermethylation within the promoter regions. In contrast, global hypomethylation contributes to genomic instability, followed by cell transformation. Meta-analyses from different labs revealed that DAPK1 promoter methylation is associated with an increased cervical cancer risk 9, 10. Methylation markers seem to be valuable tools for the early detection of cervical cancer precursor lesions. Different experimental approaches including the technique of digital loop-mediated isothermal amplification-based absolute methylation quantification revealed covincingly that hypermethylation is significantly associated with the disease severity of cervical neoplasia 11-14.

It has been shown that DAPK1 is a key modulator of cell death and autophagy, which serves an important role in the endoplasmic reticulum stress-induced cell death pathway 15. DAPK1 is a central signal transducer in different pro-apoptotic pathways that are connected with various cell death mechanisms, and is activated by various external and internal apoptotic stimuli like interferon-γ and tumor necrosis-factor-α 16. Moreover, this pro-apoptotic serine/threonine protein kinase is involved in type I apoptotic (caspase-dependent) and type II autophagic (caspase-independent) mechanisms leading to cell death 17, 18. Analysis of DAPK1 in cancer has shown that it acts as a tumor suppressor gene, which participates in tumor development and metastatic activity by regulating autophagy and apoptosis 8.

A canonical kinase domain is located at the N-terminal region of DAPK1, which is followed by a Ca^2+^/CaM autoregulatory domain that is able to suppress enzymatic activity by interacting with the catalytic cleft in the absence of CaM, thereby blocking its interaction with exogenous substrates 19. Upon CaM binding, the autoregulatory domain dissociates from the catalytic region and enables the phosphorylation of substrates 8. It has been revealed that autophosphorylation at Ser308 within the CaM autoregulatory domain decreases the affinity for CaM, thereby blocking the enzymatic activity of DAPK1 8. Towards the carboxyterminal end of DAPK1, the Ca^2+^/CaM autoregulatory domain is followed by ankyrin repeats, a Ras of complex protein (ROC)-C-terminal of ROC (COR) domain and a death domain 8. Ankyrin repeats facilitate protein-protein communications and are involved in DAPK1 degradation 20. Furthermore, the ROC-COR domain promotes GTP hydrolysis, indicating that DAPK1 is a GTP-binding protein with intrinsic GTPase activity and serves a role in cytoskeletal localization 20. This domain also contributes to homodimerization via both its kinase domain and the ROC domain. The death domain at the carboxyterminal end of DAPK1 represents a protein-protein interaction domain that can be found in numerous apoptosis-promoting proteins. Thus, DAPK1 has been considered to be involved in important pathways leading to apoptosis and autophagy.

The polo-like kinase (PLK) family of serine/threonine kinases serves a crucial role in the regulation of cell cycle progression, including the formation of bipolar spindles, chromosome segregation, centrosome maturation in late G_2_/early prophase, activation of CDK1, regulation of the anaphase-promoting complex and execution of cytokinesis 21-23. As most types of cancer cells require PLK1 for survival, in addition to the fact that PLK1 is upregulated in numerous cancer types, PLK1 has been investigated as a target for novel anticancer agents 24, 25.

At present, mechanistically different types of PLK1 inhibitors, such as ATP-competitive, non-ATP competitive and polo-box domain (PBD) inhibitors, are being examined in multiple clinical trials 6, 26-29. Currently, the most advanced ATP-competitive small molecule inhibitors are BI2536 and BI6727 (Volasertib) 18, 26, 27. The overall toxicity of PLK inhibitors is considered to be well manageable, with dose-dependent hematotoxicity as the limiting side effect in most clinically tested compounds 24, 25, 30-33.

## Materials and methods

### Antibodies and western blotting

Antibodies were used at the following dilutions: β‑actin (1:200,000, cat. no. A1978), Flag (1:1,000, cat. no. F1804 ) (all Sigma-Aldrich; Merck KGaA), DAPK (1:1,000; cat. no. ab 67286, Abcam), phosphorylated (p) histone 3 (pH3; Ser10; 1:1,000; cat. no. 06-570, MilliporeSigma), mouse pDAPK Ser308 (1:1,000; cat. no. GTX 10524, GeneTex, Inc.), mouse monoclonal anti-PLK1 (1:1,000, cat. no. sc-17783 ), CDK1 (1:1,000, cat. no. sc-54), Cyclin A (1:1,000; cat. no. sc-271682, all Santa Cruz Biotechnology, Inc.), Cyclin B1 (1:1,000, cat. no. 4138), Cyclin E (1:1,000, cat. no. 4129; all Cell Signaling Technology, Inc.), V5 (1:2,000; cat. no. 46-0705, Invitrogen). HRP-conjugated secondary antibodies (1:5,000; cat. no. 211-032-171 (rabbit), cat. no. 115-035174 (mouse), Jackson ImmunoResearch Labs, Inc.) and FITC- (1:1,000; cat. no. Ab150113), Cy3- (1:1,000; cat. no. Ab6939) and Cy5-conjugated antibodies (1:1,000; cat. no. Ab97172, all Abcam). Western blot analysis was performed as described previously 7.

### Kinase assays

Immunoprecipitation (IP) assays using DAPK1-, CDK1-, V5- or Flag-specific antibodies were performed as described previously 34. *In vitro* kinase assays were performed as described previously 34, to measure the kinase activities of DAPK1, CDK1 and PLK1, in the presence of H1/3 (New England Biolabs, Inc.) or Glutathione S-transferase (GST)-myosin light chain (MLC) 35, 36. Briefly, PLK1 was incubated with 0.5-1 μg of substrate and 2 μCi of [γ-^32^P]ATP for 20 min at 37 °C in kinase buffer (20 mM HEPES pH 7.4, 150 mM KCl, 10 mM MgCl_2_, 1 mM EGTA, 0.5 mM DTT, 5 mM NaF, 0.1 mM Na_3_VO_4_). Reactions were stopped by adding SDS sample buffer and boiling for 3 min.

### Preparation of cell culture

Cancer cell lines (HeLa, A549, SW-480, Jurkat, MCF-7, EVSA-T, MDA-MB-231, MDA-MB-468, SKOV-3) were purchased from the American Type Culture Collection, and were cultured with 10% FCS (PAA Laboratories GmbH; Cytiva) at 37 °C with 5% CO_2_ in a humidified atmosphere. MEM (Gibco) was used for culturing HeLa, A549, MDA-MB231, 468 and SKOV-3 cells. RPMI (Sigma-Aldrich; Merck KGaA) was used for culturing Jurkat, MCF-7, EVSA-T and SW480 cells.

### Patients and Samples (Primary Cell Culture)

This study was conducted according to the “REporting recommendations for tumor MARKer prognostic studies” 37. To establish primary, patient-derived cervical cancer cell cultures, we analyzed samples from patients undergoing surgical resection between January 2015 and March 2018 at the Department of Gynecology of the Goethe University Hospital in Frankfurt am Main, Germany. For the samples with validated diagnosis, sufficient archival material for immunohistochemical analysis was available. The Local Research Ethics Committees approved studies of human tissue, and samples were processed anonymously.

### Cell Culture

Primary cells were isolated from cervical cancer tissues using the tumor dissociation kit (Max Miltenyi 130-095-929) together with the tumor cell isolation kit (Max Miltenyi 130-108-339) following the manufacturer's instructions.

### Transfections

Small interfering (si)RNA was transfected into cells using Lipofectamine^®^ RNAi MAX reagent (Invitrogen; Thermo Fisher Scientific, Inc.) according to the manufacturer's protocol. For DNA transfections jetPEI from Polyplus was used according to the manufacturer's protocol.

### Cell cycle, apoptosis and cell proliferation assays

Treatment with double-thymidine or nocodazole (Noc; Sigma-Aldrich; Merck KGaA) was conducted as described previously 38. Cells were synchronized to the G_2_ phase via incubation with 5 µM RO-3306 for 16 h at 37 °C as described in a previous report 6.

For cell cycle analysis, cells were harvested, washed, fixed and stained as described previously 30. Cell cycle quantification was performed using a FACS Calibur instrument and CellQuest Pro software, Version 6.0 (both BD Biosciences).

The activity of caspase-3/7 was determined using a Caspase-Glo^®^3/7 assay (Promega Corporation), according to the manufacturer's instructions. Cell viability and proliferation assays were conducted using the Cell Titer-Blue^®^ Cell Viability assay (Promega Corporation, cat. no. G808B), as described previously 30.

### Proximity ligation assay (PLA)

PLA detection of the DAPK1/PLK1 interaction was performed using a Duolink *in situ* PLA kit (Olink Bioscience), according to the manufacturer's instructions. Briefly, it involves converting potential protein-protein interactions into DNA molecules by first targeting DAPK1 and PLK1 using specific antibodies against them, which has to be generated in two entirely different hosts. These primary antibodies are then targeted by PLA probes, each specific against the primary antibody host, conjugated with a short oligonucleotide sequence. These two oligonucleotides are then ligated using a ligase providing a template for a Rolling Circle Amplification (RCA). This template is formed only when the two proteins are located within 40 nm of each other, a distance considered to be close enough for favoring their potential interaction *in vivo*. The RCA is then amplified by a suitable polymerase and labeled with detectable probes which can then be visualized under an immunofluorescence microscope as a fluorescent dot.

### Immunofluorescence (IF) assay

For indirect IF staining, cells were seeded on cover slides. Briefly, cells were treated for the indicated time points, then fixed for 8 min with methanol containing 1% paraformaldehyde at -20 °C. The following primary antibodies were used for staining: FITC-conjugated monoclonal DAPK1 (rabbit; Cell Signaling Technology, Inc.), PLK1 (mouse; Santa Cruz Biotechnology, Inc.). FITC-, Cy5- and Cy3-conjugated secondary antibodies were obtained from Abcam, Inc. DNA was stained with DAPI (Roche Diagnostics). Images were captured using an AxioObserver.Z1 microscope with a HCX PL APO CS 63.0x1.4 oil UV objective (Carl Zeiss AG) and a confocal laser-scanning microscope (Leica CTR 6500; Leica Microsystems GmbH).

### Statistical analysis

All experiments were performed at least in triplicate. Standardization and statistics were performed with Microsoft Excel as described previously 39. For paired t-tests, all experimental groups were compared with their respective groups. P<0.05 was considered to indicate a statistically significant difference.

## Results

### DAPK1 expression during the cell cycle in cervical cancer and lung cancer cells

**Figure 1:**
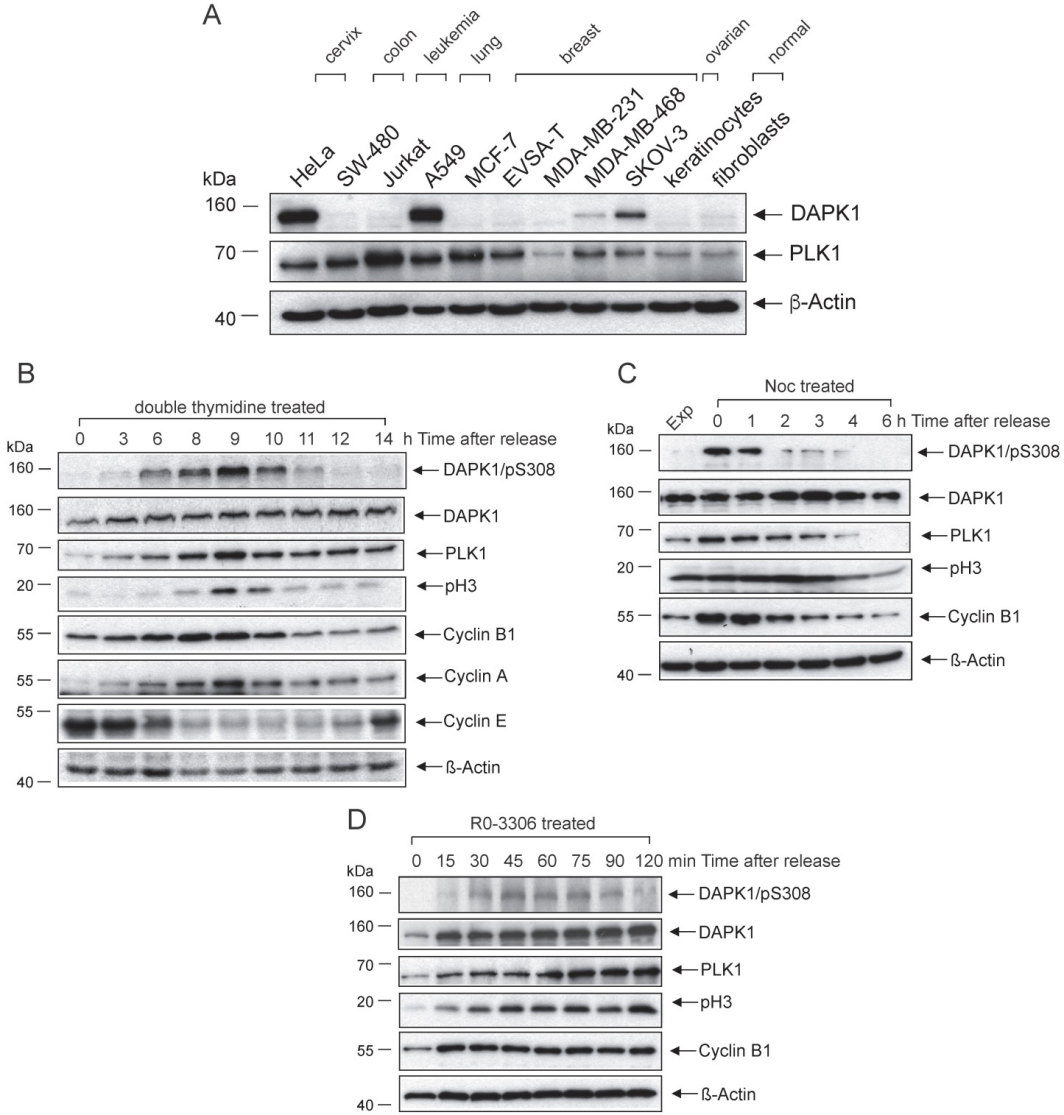
**Analyses of DAPK1 and critical regulators of the mammalian cell cycle in cervical cancer cells.** Western blot analysis of DAPK1 expression in an **(A)** panel of cancer cell lines and **(B)** HeLa cells arrested using double-thymidine. Cells were treated with **(C)** nocodazole overnight or **(D)** with the CDK1 inhibitor RO-3306. Cell lysates were immunoblotted for DAPK/pS308, DAPK1, PLK1, pH3, Cyclin B1, Cyclin A, Cyclin E and β-Actin. For all panels, one image representative of three independent experiments is shown. pH3, histone H3 phosphorylated at Ser10; p, phosphorylated; DAPK, death-associated protein kinase; PLK1, polo-like kinase 1.

Despite numerous studies examining the function of DAPK1 as a Ca^2+^/CaM-dependent serine/threonine kinase that serves a key role in multiple cellular signaling pathways, which trigger cell survival, apoptosis and autophagy 8, its roles in the mammalian cell cycle and in cervical cancer remain elusive. The current study evaluated a small range of gynecological cancer cell types (cervical, ovarian, breast) and other cancer cell types (colon, lung, leukemia), as well as primary normal cells (keratinocytes, fibroblasts) for the expression of DAPK1 via western blotting (Fig. 1A). While in most cell types DAPK1 signals were below or at the limit of detection, only the cervical HeLa cell line and the lung cancer cell line A459 showed strong DAPK1 expression. Thus, these were both used in subsequent investigations.

To examine the role of DAPK1 in the cell cycle, with a particular focus on cervical cancer cells, asynchronously growing HeLa cells with high DAPK1 expression were treated with different agents (thymidine, nocodazole, RO-3306) for synchronization. First, a method for synchronizing cells at the G_1_/S border was conducted using double treatment of thymidine, which, in excess, acts as an inhibitor of DNA synthesis. In the western blotting study, DAPK expression was observed in all stages of the cell cycle, with only moderate changes in its expression frequency (Fig. 1B). DAPK1 activity is regulated by autophosphorylation at pSer308 within the CaM-regulatory domain, which occurs at the basal state, in the absence of Ca^2+^/CaM 17. This autophosphorylation is inversely correlated with substrate phosphorylation. In double thymidine-treated cells, the DAPK/pSer308 signal started in the G_2_ phase, peaked in mitosis and decreased rapidly at mitotic exit. Monitoring of classical mitotic markers, including PLK1, Cyclin B1 and pH3, supported the observation that the inhibitory DAPK autophosphorylation at pSer308 peaks during mitosis, suggesting that the enzymatic activity of DAPK is downregulated during mitosis.

To validate the aforementioned results, additional classical agents were used for the synchronization/arrest of cells, including Noc treatment, which allows cells to enter mitosis but prevents the formation of the metaphase spindles as microtubules cannot polymerize. Thus, cells arrest in prometaphase. The western blot analysis of HeLa cells revealed a strong signal of DAPK/pSer308 in Noc-treated cells. At 1 h following the release from Noc-induced prometaphase arrest, the DAPK1/pSer308 signal began to decline (Fig. 1C). It was found that a prominent DAPK/pSer308 signal coincided with strong PLK1 and Cyclin B1 expression.

Next, the selective CDK1 inhibitor RO-3306, which arrests cells in G_2_ phase before the entry into mitosis, was used to treat cells. While under RO-3306 treatment, the DAPK/pSer308 signal was not visible, but upon release from RO-3306 the DAPK/pSer308 signal peaked within 45-60 min, shortly before peak levels of PLK1 and pH3 were detectable (Fig. 1D). Taken together, different cell cycle analyses of HeLa cells confirmed the regulation of DAPK during G_2_ and mitosis via the autophosphorylation of DAPK at pSer308.

Next, the lung cancer cell line A549, which showed high levels of DAPK protein (Fig. 1A), was used to determine whether a cell cycle-based regulation of DAPK can be detected in cell lines other than those of cervical origin. It was identified that Noc-treatment overnight arrested 73% of cells in G_2_/M (Fig. S1A). In Noc-treated cells, a prominent DAPK/pSer308 signal could be observed, which increased to maximal levels at 1 h following release and decreased strongly thereafter (Fig. S1B). In non-mitotic cells, the DAPK/pSer308 signal was not detectable. Collectively, these independent experiments using different agents for the induction of cell cycle arrest provided evidence that the autophosphorylation signal of DAPK1 at pSer308 has maximal levels during mitosis of cervical and lung cancer cells. Considering that the protein expression level of DAPK1 fluctuates only to a small extent, the regulation of DAPK1 activity during the cell cycle may be triggered at the enzymatic level and regulated by DAPK/pSer308 autophosphorylation.

### Enzymatic activity of DAPK1 during the cell cycle

**Figure 2:**
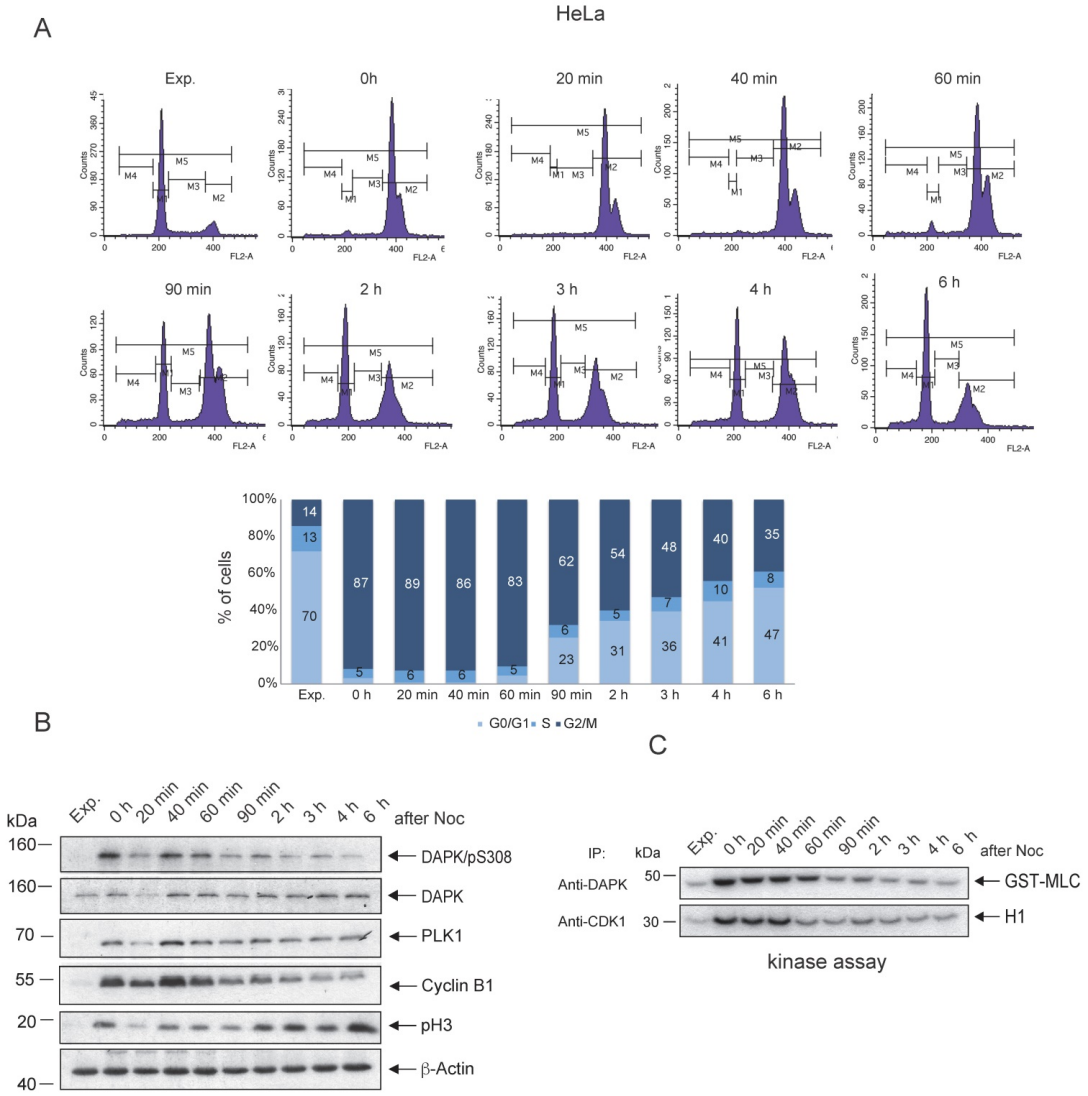
** Enzymatic activity of DAPK1 during mitosis.** (A) HeLa cells were synchronized in mitosis via nocodazole treatment, released into fresh medium and harvested at the indicated time points. FACS analyses of the cell populations at different time points following release are shown. **(B)** The accumulation of key protein markers was used to determine the cell cycle stages. Cell lysates were immunoblotted for DAPK/pS308, DAPK1, PLK1, Cyclin B1, pH3 and β-Actin. For all panels, one image representative of three independent experiments is shown. **(C)** Lysates were subjected to anti-DAPK1 or anti-CDK1 IP. Subsequently, the corresponding kinase precipitates were used for radioactive kinase assays using GST-MLC or H1 as substrates. pH3, histone H3 phosphorylated at Ser10; p, phosphorylated; DAPK, death-associated protein kinase; PLK1, polo-like kinase 1; H1, histone 1; Glutathione S-transferase (GST)-MLC, myosin light chain; IP, immunoprecipitation.

To follow up on the possible association between the peak levels of pSer308 and the enzymatic activity of DAPK1 in mitotic cancer cells, *in vitro* kinase assays of DAPK1 in comparison with the master regulator of mitosis, CDK1, were conducted. HeLa cells were treated overnight with Noc and subsequently released from Noc treatment into fresh medium. The Noc incubation of HeLa cells induced an enrichment of cells, with 87% in the G_2_/M phase (Fig. 2A). Immunoprecipitated DAPK and CDK1 from lysates of Noc-treated and released cells were subjected to kinase assays using MLC fused to GST (GST-MLC) or H1 as substrates for monitoring the catalytic activities. CDK1 from Noc-treated cells highly phosphorylated H1 (Fig. 2B, C). Upon release from Noc, strong enzymatic activity of CDK1 was detectable for ~40 min in mitosis before it faded in anaphase (Fig. 2B, C), which was in line with previous reports 40. The enzymatic activity of DAPK1 towards the exogenous substrate MLC could be measured in an extended time window, involving the entire mitosis cycle, compared with the activity of CDK1 (Fig. 2B, C). Moreover, in the G_1_ phase, the activity of DAPK was very low. The corresponding experiment using A459 lung cancer cells showed enzymatic activity of DAPK1 towards the exogenous substrate MLC for 60 min upon release from the Noc treatment (Fig. S2A-C). In summary, while DAPK is autophosphorylated at pSer308 during mitosis, which represents a regulatory mechanism that prevents cell death due to a low level of kinase activity, low levels of phosphorylation of the exogenous substrate MLC were still detectable following Noc-treatment (overnight) and for 60 min upon release from the pro-/metaphase arrest in cervical and lung cancer cells.

### Localization and interaction of DAPK1 and PLK1 during mitosis

**Figure 3:**
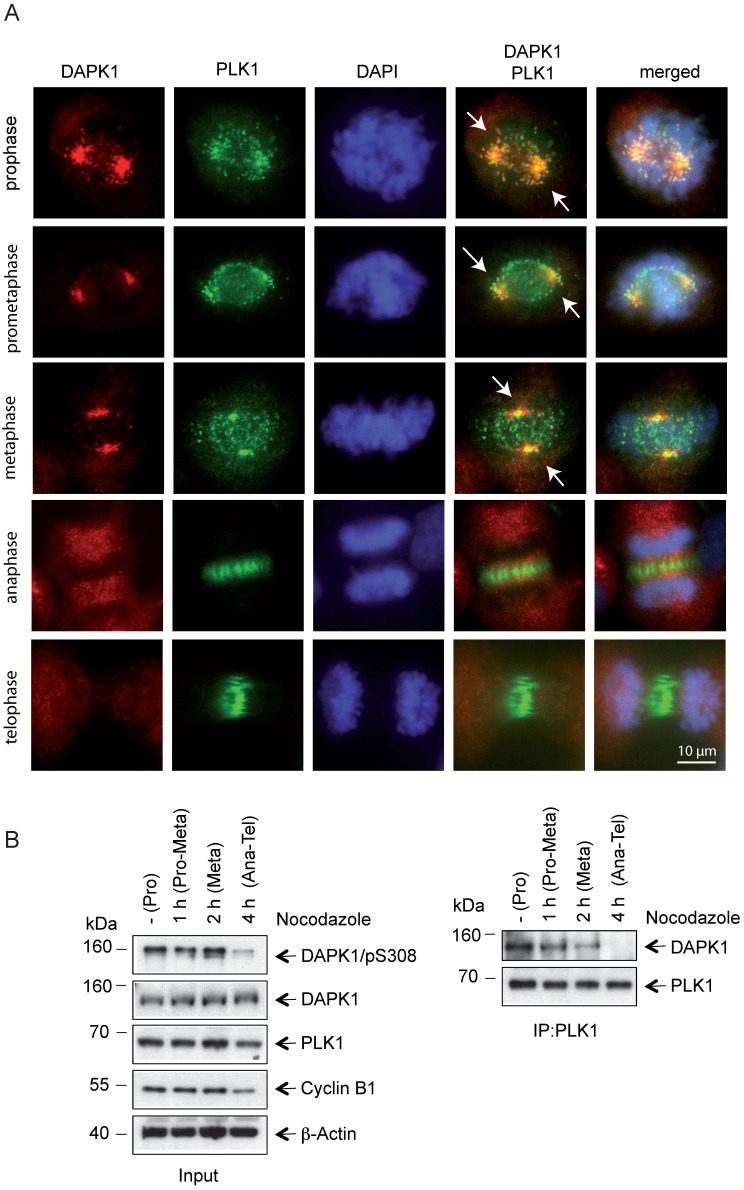
** Co-localization of PLK1 and DAPK1 during mitosis. (A)** Nocodazole-treated cells were fixed and processed for immunofluorescence using antibodies for DAPK1 and PLK1. DNA was stained with DAPI. Arrows highlight the co-localization of DAPK1 and PLK1 at and close to centrosomes. Scale bar, 10 μm. **(B)** Hela cells were treated overnight with nocodazole and released for the indicated time periods. For the co-immunoprecipitation experiment, PLK1 was immunoprecipitated and analyzed via western blotting for DAPK1. DAPK, death-associated protein kinase; PLK1, polo-like kinase 1.

Profound alterations in cell physiology are hallmarks of mitosis. These functional changes are accompanied by enhanced protein phosphorylation caused by a large portion of the kinome, predominately serine/threonine kinases, including classic mitotic kinases, such as CDKs, Aurora kinases, PLKs, never in mitosis in A (NIMA)-related kinases (NEKs), haspin and greatwall kinase 41. Collectively, these kinases allow for the correct timing and dependability of processes encompassing centrosomal functions, spindle assembly and microtubule-kinetochore attachment, as well as sister chromatid separation and cytokinesis 41. To monitor the mitotic functions of DAPK in this context in further detail, IF microscopy of mitotic cells was performed and DAPK1 was detected predominately at centrosomes from prophase to metaphase (Fig. 3A). Centrosomes contain a structured centriole pair surrounded by an amorphous material known as the pericentriolar material (PCM) 42. During mitotic exit centrosomes change their phenotype, which is a characteristic for centrosome disassembly 43. The IF staining results suggested that DAPK1 was associated with the PCM in late stages of mitosis.

Recent evidence regarding the molecular basis in centrosome strength and ductility in *C. elegans* embryos revealed that first steps of centrosome disassembly were correlated with inactivation of PLK1 and spindle-defective protein-2 (Centrosomal Protein 192 in humans) 44. To investigate a putative functional correlation of PLK1 and DAPK1, the localization of PLK1 during mitosis was examined and it was demonstrated that both kinases colocalized from prophase until metaphase (Fig. 3A). In anaphase, PLK1 disassociated from centrosomes and a change to a weak state of centrosomes occurred, which enabled a force-mediated centrosome disassembly. The dissociation of PLK1 from compact centrosomes coincided with a loss of DAPK1/PLK1 co-localization. The IP of endogenous protein using PLK1 antibodies revealed a direct interaction between PLK1 and DAPK1 in metaphase (Fig. 3B). Furthermore, an interaction between both these proteins started to be visible at 1-2 h before metaphase.

**Figure 4:**
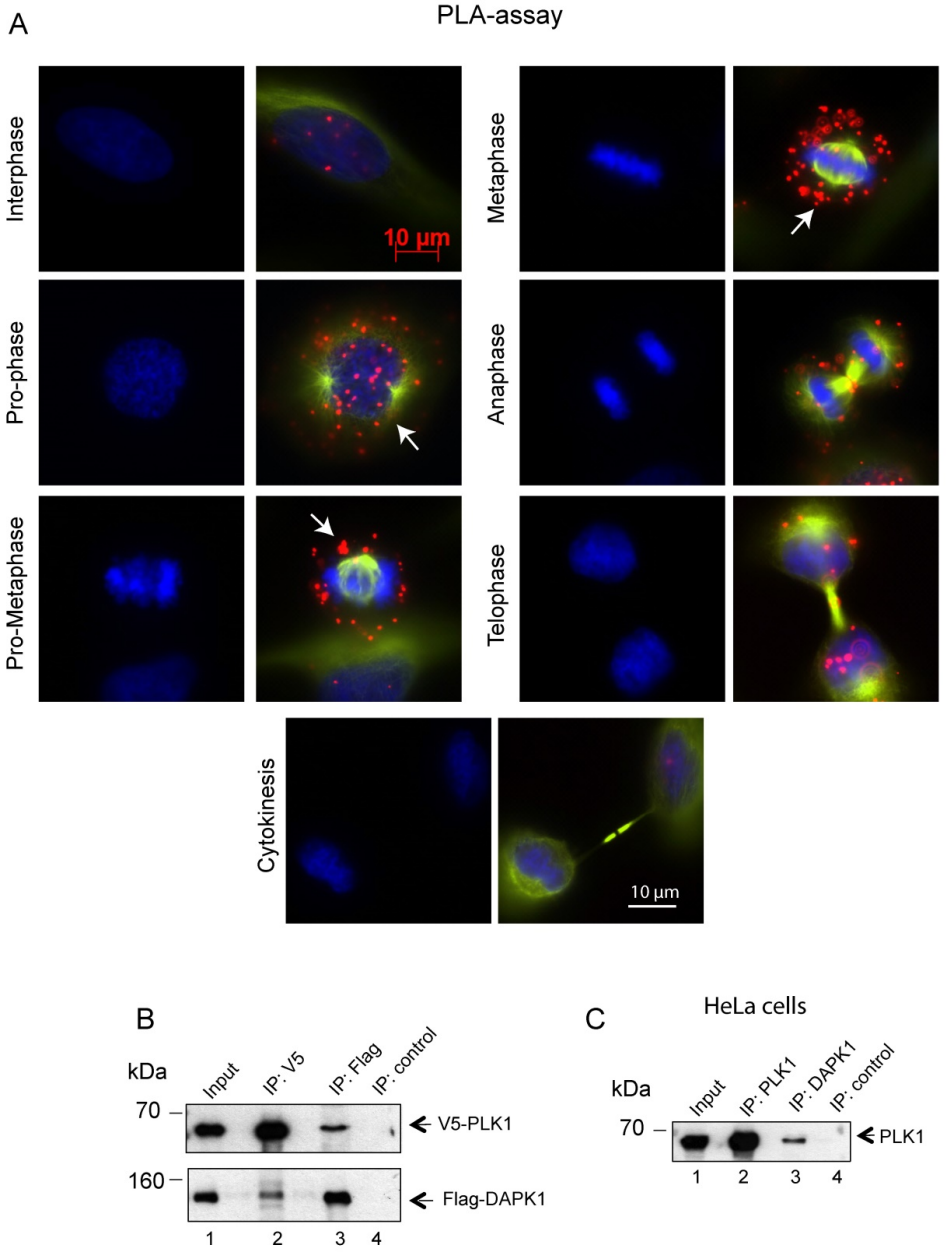
**Assessing the co-localization of PLK1 and DAPK1 during mitosis using the proximity ligation assay. (A)** *In situ* PLA detection of DAPK1 and PLK1 in nocodazole-arrested cells. Red dots indicate interactions between DAPK1 and PLK1. Arrows highlight the co-localization of DAPK1 and PLK1 in the vicinity of centrosomes. Scale bar, 10 µm. **(B)** The interaction between PLK1 and DAPK1 was investigated in HeLa cells overexpressing V5-PLK1 and Flag-DAPK via co-immunoprecipitation using V5- or Flag-specific antibodies. The precipitates and 5% of the input were subjected to immunoblotting using the indicated antibodies. DAPK, death-associated protein kinase; PLK1, polo-like kinase 1. **(C)** The lysates of HeLa cells were subjected to IP using PLK1- or DAPK1-specific antibodies followed by western blotting with anti-PLK1 antibodies.

Subsequently, the interaction between endogenous DAPK1 and PLK1 in cervical cancer cells was visualized using the PLA, which depends on recognition of target proteins by pairs of antibody-oligonucleotide conjugates (Fig. 4A). Since one dot in the PLA corresponds to one co-localization, it was concluded that the interaction occurred predominately from prophase to metaphase. The association of DAPK1 and PLK1 appeared to be much weaker in late stages of mitosis and was absent in interphase (Fig. 4A), thus supporting previous IF experiments (Fig. 3A). To follow up on the hypothesis that there may be a direct interaction between PLK1 and DAPK1, V5-tagged PLK1 and Flag-DAPK1 were co-expressed in cells and a co-IP assay was performed. Interestingly, the PLK1-IP assay revealed the presence of DAPK1 that complexed with PLK1 in cells (Fig. 4B). The reverse co-IP, using Flag-DAPK1, also supported the interaction of both proteins. In addition, we precipitated endogenous PLK1 from HeLa cells and identified DAPK1 in the precipitate using DAPK1-specific antibodies (Fig. 4C) corroborating the previous IP results using recombinant protein (Fig. 4B).

**Figure 5:**
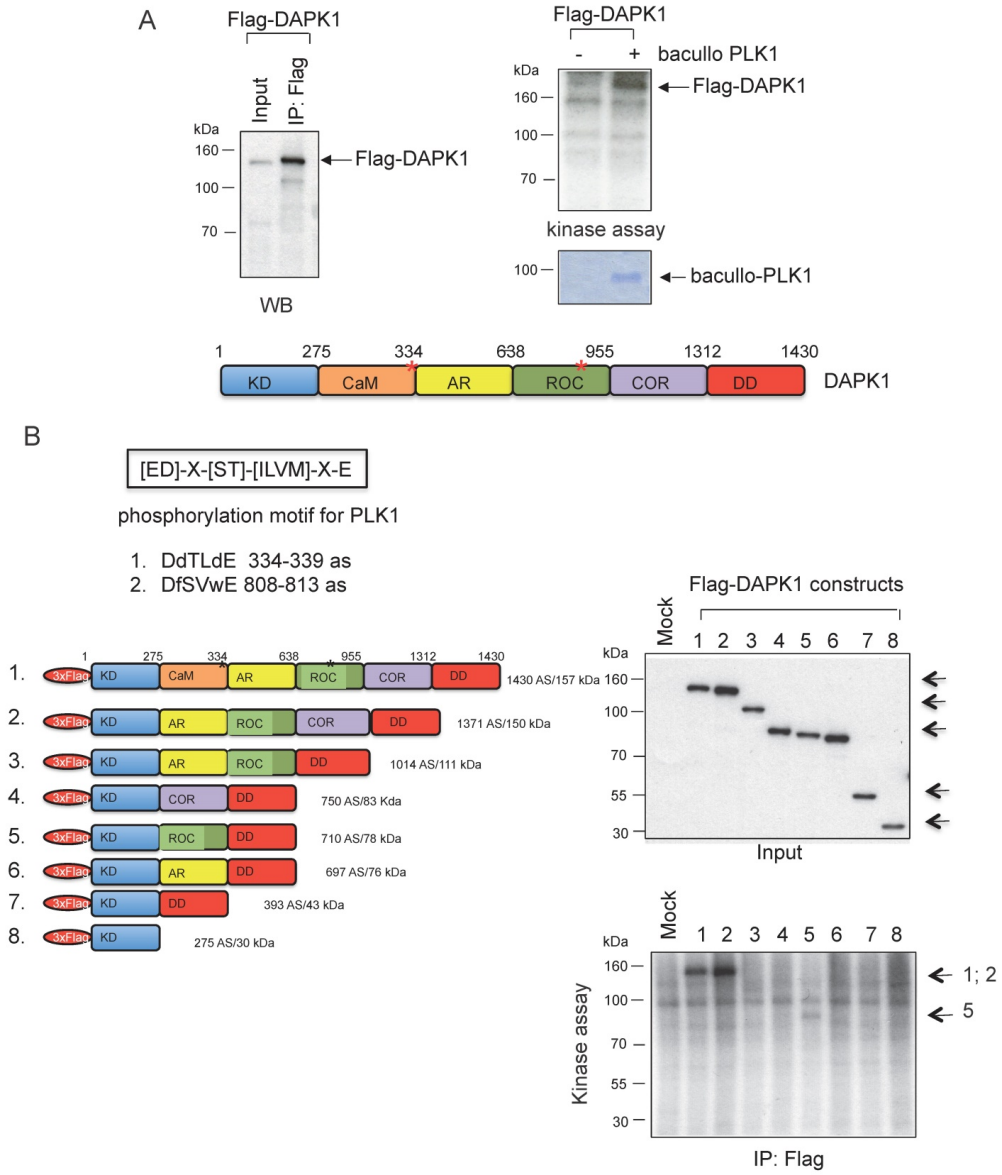
** DAPK1 is a substrate of PLK1. (A)** Recombinant Flag-DAPK1 expressed in 293T cells was immunoprecipitated using Flag-specific antibodies and then subjected to kinase assays, including γ-^32^P ATP and PLK1 expressed in SF9 insect cells. (B) Functional domains of DAPK1, including PLK1 consensus motifs with DAPK1, are depicted: KD, Ca^2+^/CaM autoregulatory domain, AR, the ROC-COR domain and the DD. Lower left: Full-length DAPK (1) and a panel of DAPK1 deletions clones (2-8). Upper right: Full-length DAPK and its deletions clones (2-8) were separated via PAGE, and lower right: Were subjected to a radioactive protein kinase assay, including PLK1 from SF9 cells. KD, kinase domain; Ca^2+^/CaM, calcium/calmodulin; AR, ankyrin repeats; ROC, Ras of complex protein; COR, C-terminal of ROC; DD, death domain; DAPK, death-associated protein kinase; PLK1, polo-like kinase 1.

Moreover, kinase assays were performed to determine whether DAPK1 and/or PLK1 act as substrates for their corresponding partner. To this end, Flag-DAPK1 was expressed in mammalian cells, DAPK1 was immunoprecipitated and purified PLK1 was added from bacullo SF9 cells (Fig. 5A). DAPK1 was found to be a substrate for PLK1 (Fig. 5A, right panel). A computer-based search was used to identify the phosphorylation sites of PLK1 in the protein sequence of DAPK, and two PLK1 sites were found: One of the consensus motifs was found at the amino-terminal end of the Ca^2+^/CaM domain and a second motif within the ROC domain (Fig. 5B, upper panel). To more closely determine putative phosphorylation sites of PLK1 with DAPK1, several deletion clones of DAPK1 (clones 1-8) were generated (Fig. 5B, lower left panel). While clones 1 and 2 showed strong phosphorylation signals in radioactive kinase assays, clone 5, which was missing the CaM domain, could only be weakly phosphorylated (Fig. 5B, lower right panel), thus supporting the notion of the phosphorylation of DAPK1 at different sites by PLK1. Since clone 2 was also missing the CaM domain, the phosphorylation event may occur within the ROC domain. Despite this, the CaM domain should also be considered as putative region of phosphorylation.

### DAPK1 contributes to the death of cervical cancer cells induced by topotecan

**Figure 6:**
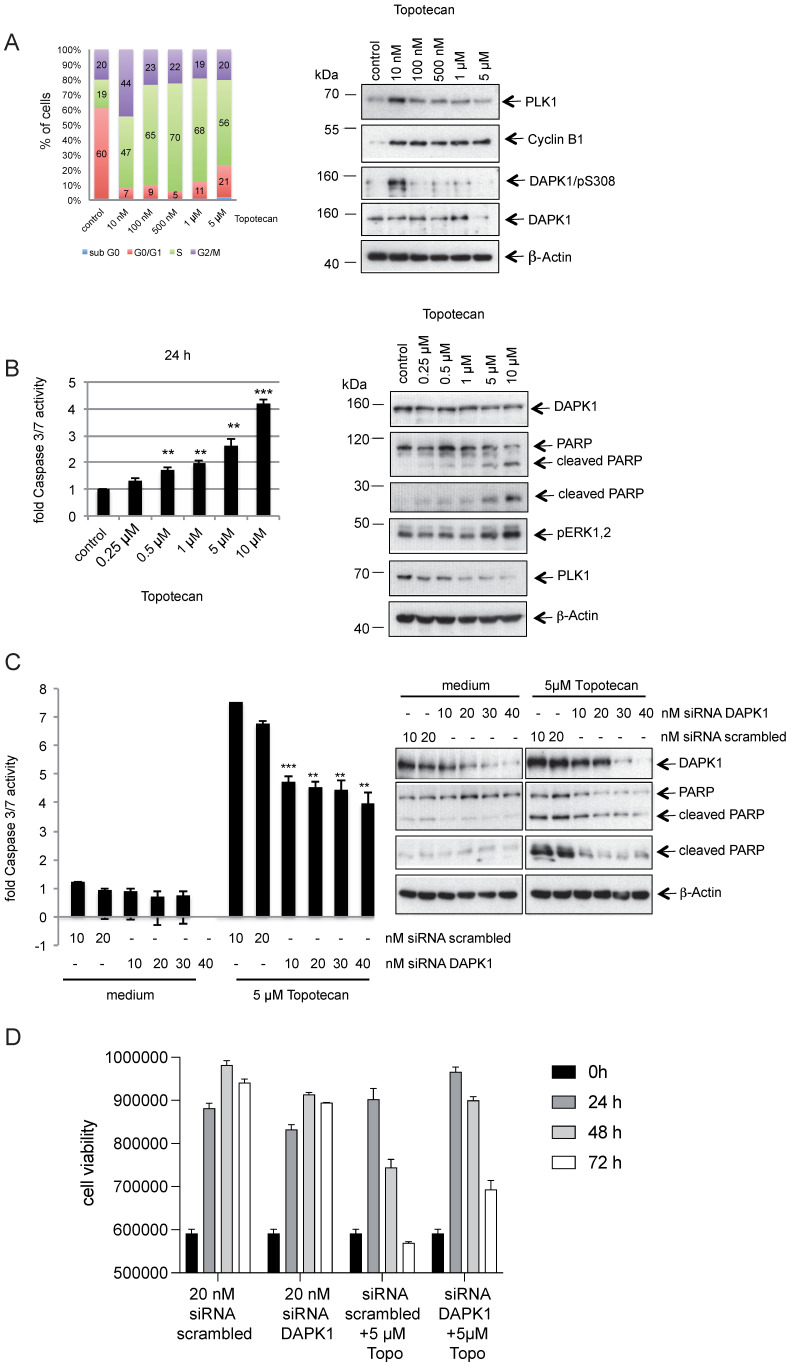
** Topotecan treatment and the role of DAPK1 in HeLa cells. (A)** (Left) HeLa cells were treated with different concentrations of topotecan and analyzed via FACS. (Right) Lysates were immunoblotted for PLK1, Cyclin B1, DAPK/pS308, DAPK1 and β-Actin. **(B)** Concentration-dependent, apoptotic response of HeLa cells. (Left) Caspase-3/7 activity was determined in the lysates of cells treated with increasing concentrations of topotecan using the Caspase-Glo^®^ 3/7 assay (mean values of three independent experiments for each concentration). DMSO was used as the control treatment. (Right) Lysates were immunoblotted for DAPK1, PARP, pERK1/2, PLK1 and β-Actin. **(C)** Treatment of DAPK1-depleted cancer cells with topotecan. (Left) HeLa cells were transfected with siRNA scrambled as control, or siRNA DAPK1. Caspase-3/7 activity was determined using the Caspase-Glo 3/7 assay. ^*^P<0.05, ^**^P<0.01, ^***^P<0.001. Student's t-test, unpaired and two-tailed. (Right) Lysates were harvested and analyzed via immunoblotting for DAPK1, PARP and β-Actin. For all panels, one image representative of three independent experiments is shown. DAPK, death-associated protein kinase; PLK1, polo-like kinase 1; p, phosphorylated; IP, immunoprecipitation; PARP, poly(ADP-ribose) polymerase; siRNA, small interfering RNA. **(D)** HeLa cells were treated with 20 nM siRNA scrambled or 20 nM DAPK1-specific RNA with of without 5 μM topotecan and cell viability was measured over 3 days using the Cell Titer-Blue^®^ Cell Viability Assay.

DNA topoisomerase I is an enzyme that mitigates the torsional stress of DNA by generating transient single-strand breaks 45. Small molecule inhibitors of DNA topoisomerase I are clinically used to treat ovarian, small cell lung and cervical cancer types 46. To study the role of DAPK1 and its phosphorylation at pSer308 in the response to topotecan, HeLa cells were treated with increasing concentrations (10 nM - 5 μM) of the camptothecin-derivative topotecan and the cell cycle profile was analyzed (Fig. 6A). While topotecan at a low concentration (10 nM) induced an enrichment of HeLa cells with 44% in the G_2_/M phase compared with control cells (only 20% in the G_2_/M phase), it was found that higher concentrations, such as 500 nM, had a more profound effect on cells, with 70% in the G_1_/S phase (Fig. 6A, left panel). The autophosphorylation of DAPK at pSer308 was only detectable when HeLa cells were treated with 10 nM topotecan, inducing a moderate mitotic enrichment (Fig. 6A, right panel), as previously observed using different agents for inducing cell arrest (Fig. 1A-C). At higher concentrations of topotecan, which resulted in a high percentage of cells in the G_1_/S phase, the pSer308 signal was barely visible (Fig. 6A, right panel).

The western blotting results demonstrated an inverse association between increasing concentrations of topotecan and the PLK1 signal (Fig. 6A, right panel), which is a marker for the proliferative activity of cells 26, 27. Moreover, increasing concentrations of topotecan induced rising levels of intrinsic apoptosis, as indicated by the levels of caspase-3/7 activity (Fig. 6B, left panel). Poly(ADP-ribose) polymerase 1 (PARP-1) is one of several known cellular substrates of caspases 47. Thus, PARP cleavage upon topotecan treatment in HeLa cells was examined. As expected, an upregulation of PARP cleavage was associated with rising levels of caspase-3/7 activity (Fig. 6B). Next, DAPK1 was knocked down using RNA interference (RNAi) and cell death activity was measured to investigate the role of DAPK1 in the topotecan-induced death of HeLa cells. Knockdown of cellular DAPK1 expression was associated with a significant decrease of intrinsic cell death in topotecan-treated cells, as indicated by the reduced caspase-3/7 activities and PARP cleavage, suggesting an important role of DAPK1 in the death of cervical cancer cells induced by topotecan (Fig. 6C). Cell viability assays confirmed reduced cell death in topotecan-treated HeLa cells upon RNAi-based depletion of DAPK1 (Fig. 6D).

**Figure 7:**
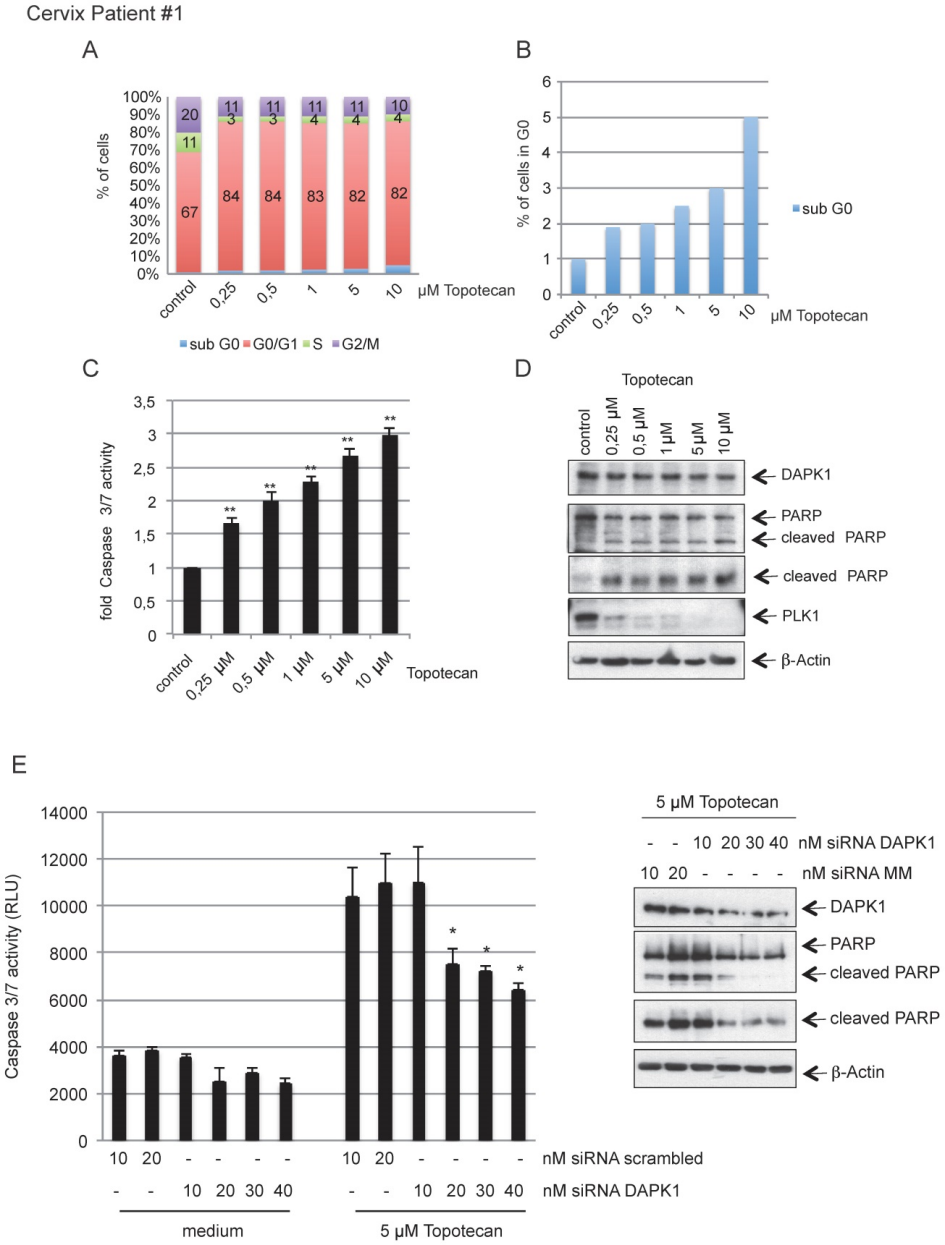
** Topotecan treatment and the role of DAPK1 in primary cervical cancer cells. (A)** Primary cervical cancer cells were treated with different concentrations of topotecan and analyzed via FACS. **(B)** Concentration-dependent, apoptotic response of primary cells. Sub-G_0_ levels were determined by FACS and **(C)** Caspase-3/7 activity was determined in lysates of cells treated with increasing concentrations of topotecan using the Caspase-Glo^®^ 3/7 assay (mean values of three independent experiments for each concentration). DMSO was used as the control treatment. **(D)** Lysates were immunoblotted for DAPK1, PARP, PLK1 and β-Actin. **(E)** Treatment of DAPK1-depleted primary cells with topotecan. (Left) Cells were transfected with siRNA scrambled as control, or siRNA DAPK1. Caspase-3/7 activity was determined using the Caspase-Glo 3/7 assay. ^*^P<0.05, ^**^P<0.01. Student's t-test, unpaired and two-tailed. (Right) Lysates were harvested and analyzed via immunoblotting for DAPK1, PARP and β-Actin. For all panels, one image representative of three independent experiments is shown. DAPK, death-associated protein kinase; PLK1, polo-like kinase 1; PARP, poly(ADP-ribose) polymerase; siRNA, small interfering RNA.

To investigate the clinical relevance of our findings, we isolated primary cervical cancer cells from surgical samples obtained from in-house surgery and treated cells with increasing concentration of topotecan (Fig. 7A) inducing rising levels of intrinsic apoptosis, as indicated by the levels of cells in sub-G_0_ and caspase-3/7 activity (Fig. 7B, C). The western blotting results revealed an inverse association between increasing concentrations of topotecan and PLK1 as marker of cellular proliferation accompanied by increased levels of cleaved PARP (Fig. 7D). Reducing endogenous levels of DAPK1 by RNAi reduced caspase-3/7 activity and also downregulated PARP cleavage (Fig. 7E). A second representative sample of cervical cancer shown in Fig. S3A-E supports the model that topotecan-induced cell death involves the function of DAPK1 in cervical cancer cells.

## Discussion

Members of the DAPK family are involved in various apoptotic, necrotic and autophagic cell processes 8. DAPK1 is a Ca^2+^/CaM-regulated serine/threonine protein kinase involved in the regulation of cancer cells from diverse origins. The inhibition of DAPK function in several, but not all, human cancer malignancies has been mainly attributed to the deregulation of epigenetic control. The key mechanisms encompass DNA hypermethylation, deacetylation of core histone proteins and RNAi. Hypermethylation has been frequently observed in various gynecological cancer types, including ovary 48, breast 49, and, in particular, cervical cancers, as reported recently by a comprehensive meta-analysis 10. This systematic meta-analysis revealed that DAPK1 promoter methylation was associated with an increased cervical cancer risk. Additional mechanisms for the silencing of DAPK1 function include allelic loss in the region of the DAPK gene in non-small cell lung cancer cell lines 50 and post-translational mechanisms, such as the hyperphosphorylation of the DAPK protein in colon cancer cell lines and primary tissues with high SRC activity 36. Although DAPK serves an important role in a wide array of apoptotic signals, including IFN-γ, TNF-α, Fas, activated c-Myc and detachment from extracellular matrix 8, its proapoptotic role in certain tumor types, such as cervical cancer, remains elusive. To this end, the current study examined the expression and post-translation modifications of DAPK1 in cervical cancer cells.

In this study we demonstrated that DAPK1 was expressed throughout the cell cycle. The detection of the inhibitory autophosphorylation of DAPK1 at pSer308 was restricted to G_2_ phase and mitosis, which suggests a safeguard mechanism to protect the fidelity of chromosome segregation during cell division. Interestingly, upon overnight Noc-treatment, weak substrate phosphorylation (MLC) was detected, indicating that, despite the inhibition of kinase activity by Ser308 phosphorylation, a basal level of extrinsic activity was maintained, which was below the threshold for the induction of apoptosis. To determine the role of DAPK1 during mitosis, the present study investigated its localization in comparison with kinases that are key players in mitotic signaling. Notably, in the early stages of mitosis the IF results demonstrated the co-localization of DAPK1 and PLK1, a serine/threonine kinase with peak enzymatic activity during mitosis. To validate the hypothesis of a putative interaction between DAPK1 and PLK1, proximity ligation assays and IPs of recombinant kinases using different antibodies were performed that could corroborate the association of both proteins. Interestingly, DAPK1 was found to be a substrate of PLK1, thus supporting the notion that during mitosis DAPK1 is phosphorylated at least by two protein kinases, namely itself and PLK1, for the regulation of protein function. Despite these findings, how PLK1 binds to DAPK1 remains to be elucidated. The PBD of PLK1 serves a crucial role in its interaction with substrates and for its subcellular localization 51, 52. Different reports have provided evidence that the PBD binds to a phosphorylated residue generated by PLK1 itself (self-priming) or by a pro-directed kinase, such as CDK1 (non-self-priming) 29, 53, 54. Thus, both protein kinases (PLK1, CDK1) should be evaluated for their ability to prime DAPK1 for the binding of PLK1.

It was interesting to observe the co-localization of DAPK1 and PLK1 in early stages of mitosis at the centrosome, which represents the major microtubule-organizing center for cytoskeleton maintenance during interphase and for mitotic spindle assembly in mammalian cells. The centrosome, as supramolecular protein complex, duplicates once per cell cycle and each duplicated centrosome represents a spindle pole that supports the fine-tuned orchestration of chromosome separation, including the maintenance of genomic stability 55. Numerous studies have reported the contribution of mitotic kinases, including NEKs, CDKs, PLKs and Aurora kinases, to regulate this precise cellular process 56. While the role of PLK1 in centrosome maturation, wherein it phosphorylated components of the PCM, leading to the accumulation of PCM, including γ Tubulin ring complex recruitment factors at centrosomes, has been discussed in detail 39, the role of DAPK at centrosomes remains unknown. While PLK1 and DAPK1 co-localize, centrosomes are visible as condensed protein complexes. Once PLK1 dissociates from centrosomes, DAPK1 remains associated with the decondensed PCM. Considering that PLK1 may phosphorylate DAPK1 within its ROC domain, PLK1 could contribute to the regulation of DAPK1 localization during mitosis. Depending on the context, increased activity of the Ras/Raf/MAPK/ERK pathway induces activation or inactivation of DAPK. ERK interacts with the death domain of DAPK, followed by the phosphorylation of Ser735 57, which leads to increased enzymatic activity of DAPK towards MLC. This enhancement appears to be affected by improved substrate binding upon Ser735 phosphorylation. Thus, it should also be considered that the phosphorylation of both putative sites within DAPK1 by PLK1 help to downregulate the catalytic activity of DAPK, thereby acting as a safeguard mechanism for the fine-tuned execution of mitosis.

Since topotecan is one of the most effective drugs for the treatment of cervical cancer and because its combination with cisplatin is effective as second-line chemotherapy in patients with advanced/recurrent cervical cancer 58, the current study examined the role of DAPK1 in the response to topotecan. While cancer cells are often protected from undergoing death receptor-mediated apoptosis by active ERK1/2 (pERK 1/2) 59, the role of ERK1/2 in DAPK-expressing cervical cells appears to be different. DAPK was identified to interact with ERK 57. Moreover, phosphorylation of DAPK at Ser735 by ERK increases the enzymatic activity of DAPK *in vivo*. The present study demonstrated that treating cervical cancer cells with topotecan caused increased levels of cells death, which was associated with increasing levels of active pERK1/2, thereby suggesting that topotecan-promoted cells death was at least partially mediated by the activation of DAPK1 via ERK1/2 activity. Notably, the downregulation of DAPK1 by RNAi reduced the apoptotic response to topotecan in cervical cancer cell lines and in primary cervical cancer samples, indicating that DAPK contributes to cell death signaling under topotecan treatment. This observation could be in line with the induction of cell death induced by ERK1/2-mediated DAPK phosphorylation.

In conclusion, a significant association between the methylation of DAPK1 promoter which correlates inversely with the expression level of DAPK1 and cervical cancer malignancy was demonstrated previously suggesting an important role of DAPK1 for the aggressiveness of cervical cancer. Considering the current observation in cervical cancer cells regarding the association between DAPK levels and the response to topotecan, the reactivation of DAPK1 in cervical tumors that do not express DAPK by demethylating agents may re-sensitize cervical cancer to topotecan and other chemotherapeutics. Furthermore, the levels of DAPK could be a valuable biomarker in predicting the outcomes of women with cervical cancer treated with chemotherapeutics, such as topotecan.

## Conclusions

Cervical cancer is one of the most common cancers among women worldwide with more than 500,000 new cases in 2021. In the present study, we demonstrate for the first time that the kinase activity of the death-associated protein kinase 1 (DAPK1) is regulated in a cell cycle-specific manner in cervical cancer cells. Furthermore, our data revealed that DAPK1 localizes to centrosomes, that orchestrate the separation of chromosomes during cell division. Remarkably, DAPK1 co-localizes and interacts with polo-like kinase 1 (PLK1) during mitotic progression. DAPK1 was found to be a substrate of PLK1. Topotecan is an effective drug used for the treatment of cervical cancer. Inhibition of DAPK1 activity by RNA interference decreased the apoptotic effect of topotecan. Our study suggests that DAPK1 could be a biomarker and a potential target for the response to topotecan during the therapy of patients with cervical cancer.

## Supplementary Material

Supplementary figures.
