# Supplementary material for: The Role of DAPK1 in the Cell Cycle Regulation of Cervical Cancer Cells and in Response to Topotecan
Source: J Cancer. 2022 Jan 1;13(3):728–43. doi: 10.7150/jca.66492 (PMC8824877; doi:10.7150/jca.66492)

Supplementary figure legends

Figure S1. Analyses of DAPK1 and critical regulators of the mammalian cell cycle in lung cancer cells. (A) FACS analysis and (B) Western blot analysis of A549 cells arrested using nocodazole treatment overnight. Cell lysates were immunoblotted for DAPK/pS308, DAPK1, PLK1, pH3, Cyclin B1 and β-Actin. For all panels, one image representative of three independent experiments is shown. DAPK, death-associated protein kinase; PLK1, polo-like kinase 1; pH3, histone H3 phosphorylated at Ser10; p, phosphorylated; cyclin B1 and ß-Actin.

Figure S2. Enzymatic activity of DAPK1 during mitosis of lung cancer cells. (A) A549 cells were synchronized in mitosis using a nocodazole treatment, released into fresh medium and harvested at the indicated times. FACS analyses of the cell populations at different time points following release are shown. (B) The accumulation of key protein markers was used to determine the cell cycle stages. Cell lysates were immunoblotted for DAPK1/pS308, DAPK1, PLK1, Cyclin B1, pH3 and β-Actin. For all panels, one image representative of three independent experiments is shown. (C) Lysates were subjected to anti-DAPK1 or anti-CDK1 IP. Subsequently, the corresponding kinase precipitates were subjected to radioactive kinase assays, using GST-MLC or H1 as substrates. pH3, histone H3 phosphorylated at Ser10; p, phosphorylated; DAPK, death-associated protein kinase; PLK1, polo-like kinase 1; H1, histone 1; GST-MLC, Myosin light chain; IP, immunoprecipitation.

Figure S3. Topotecan treatment and the role of DAPK1 in primary cervical cancer cells. (A) Primary cervical cancer cells were treated with different concentrations of topotecan and analyzed via FACS. (B) Concentration-dependent, apoptotic response of primary cells. Sub-G_0 levels were determined by FACS and (C) Caspase-3/7 activity was determined in lysates of cells treated with increasing concentrations of topotecan using the Caspase-Glo® 3/7 assay (mean values of three independent experiments for each concentration). DMSO was used as the control treatment. (D) Lysates were immunoblotted for DAPK1, PARP, PLK1 and β-Actin. (E) Treatment of DAPK1-depleted primary cells with topotecan. Cells were transfected with siRNA scrambled as control, or siRNA DAPK1. Caspase-3/7 activity was determined using the Caspase-Glo 3/7 assay. $^{*}P<0.05$, $^{**}P<0.01$. Student's t-test, unpaired and two-tailed. Lysates were harvested and analyzed via immunoblotting for DAPK1, PARP and β-actin. For all panels, one image representative of three independent experiments is shown. DAPK, death-associated protein kinase; PARP, poly(ADP-ribose) polymerase; siRNA, small interfering RNA.

A549

Suppl.Figure 1

A

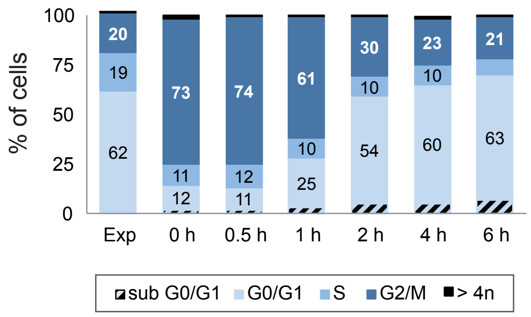

B

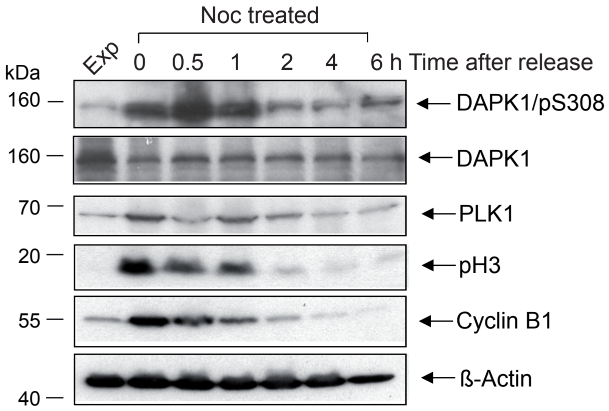

Suppl.Figure 2

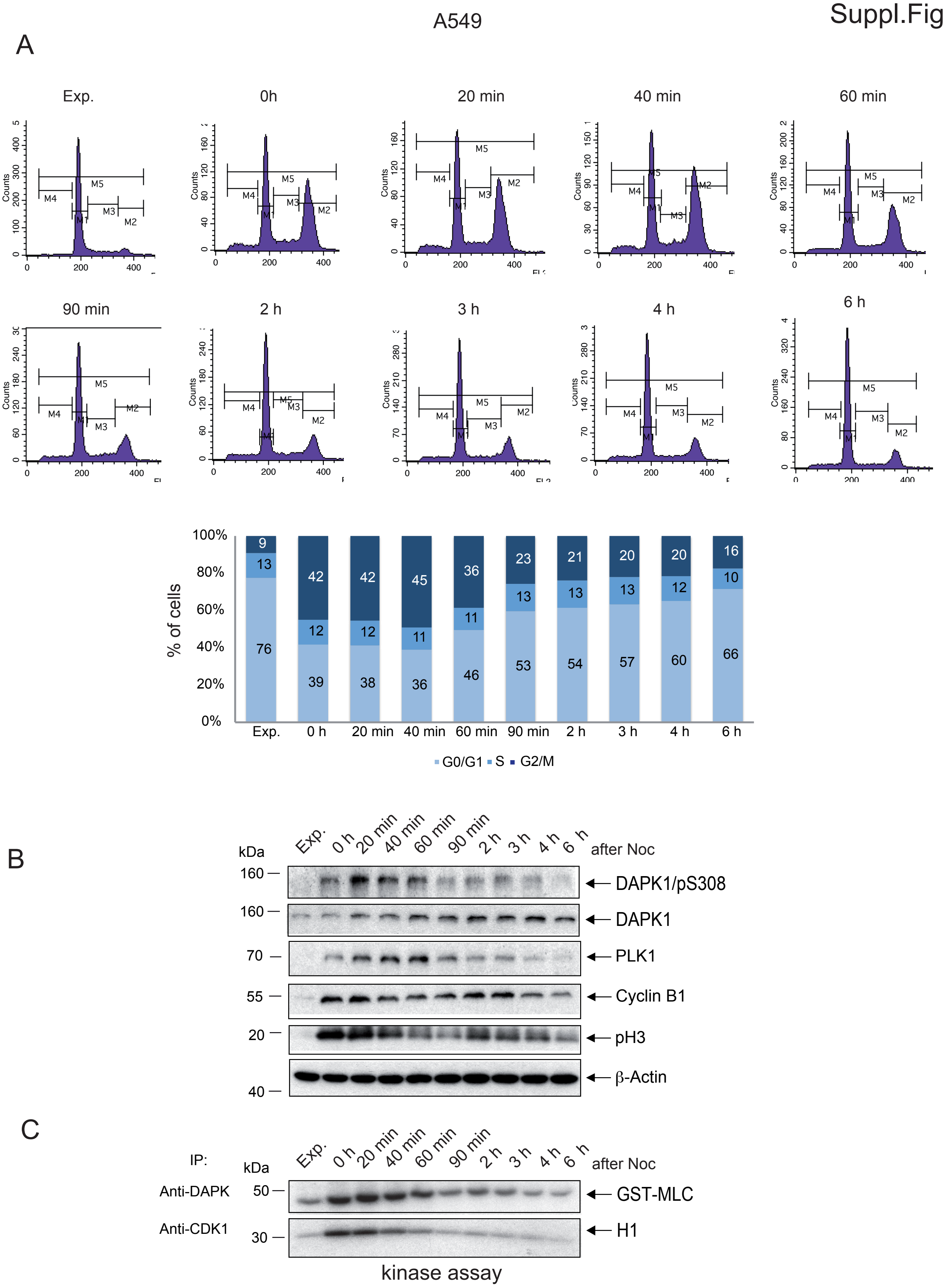

Cervix Patient #2

Suppl.Figure 3

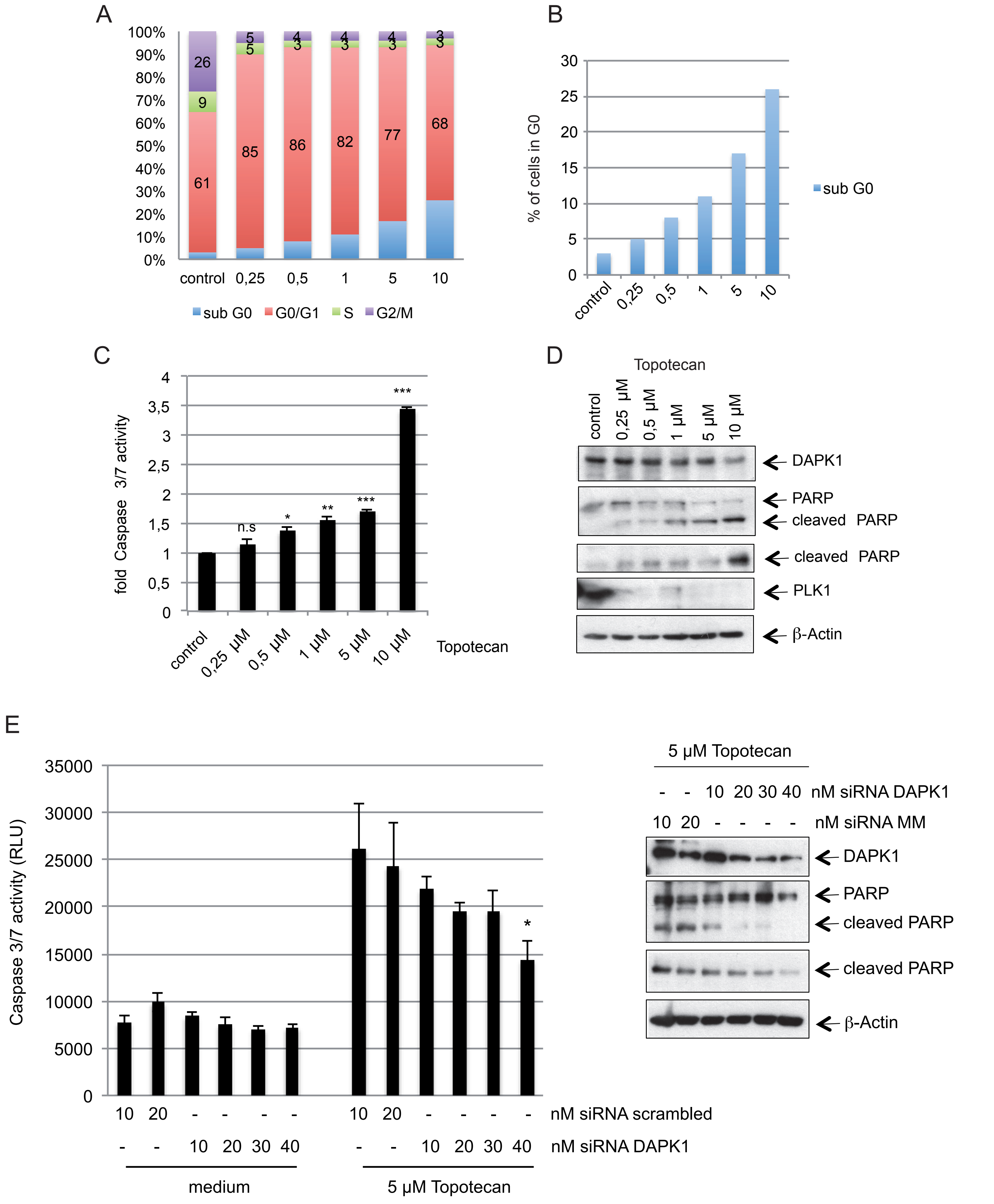

Supplement: Supplementary file 1 — Supplementary figures. [file jcav13p0728s1.pdf]
